# Optical coherence tomography with enhanced contrast using oriented magnetic nanorods

**DOI:** 10.1007/s12200-025-00167-1

**Published:** 2025-12-05

**Authors:** Seyyede Sarvenaz Khatami, Mohammad Ali Ansari, Behnam Shariati Bein Kalaee, Valery V. Tuchin

**Affiliations:** 1 Laser and Plasma Research Institute, Shahid Beheshti University, Tehran, 19839 69411 Iran; 2 Science Medical Center, Saratov State University, Saratov, 410012 Russia; 3 Institute of Precision Mechanics and Control, Federal Research Center “Saratov Scientific Center of the Russian Academy of Sciences”, Saratov, 410028 Russia

**Keywords:** Magnetic nanorods, Optical coherence topography (OCT), Ultrasound wave, Contrast-to-noise ratio (CNR), Signal-to-noise ratio (SNR)

## Abstract

In recent years, the utilization of nanoparticles with varying morphologies in optical coherence tomography (OCT) has gained prominence, primarily aimed at enhancing imaging contrast and depth. Various factors associated with nanoparticles, encompassing their shape, orientation, and distribution within biological tissues, significantly influence OCT performance. A thorough investigation of these parameters has yielded substantial findings, particularly regarding the enhancement of OCT images facilitated by the presence of nanorods (NRs). In this study, we conducted OCT imaging of chicken breast tissue employing Fe_3_O_4_ NRs under different polarization states, utilizing solenoids to apply a magnetic field to the nanoparticles. The results demonstrate that orienting nanoparticles can improve the Contrast-to-Noise Ratio (CNR) and signal-to-noise ratio (SNR) of OCT signal more than twofold compared to scenarios lacking specified orientation. Furthermore, this article addresses the challenge of prolonged nanoparticle distribution in tissue when using ultrasound probes, successfully reducing the distribution time from approximately 45 min to about 5 min. The findings presented herein show significant promises for advancing optical coherence tomography across a variety of applications.

**Graphical Abstract:**

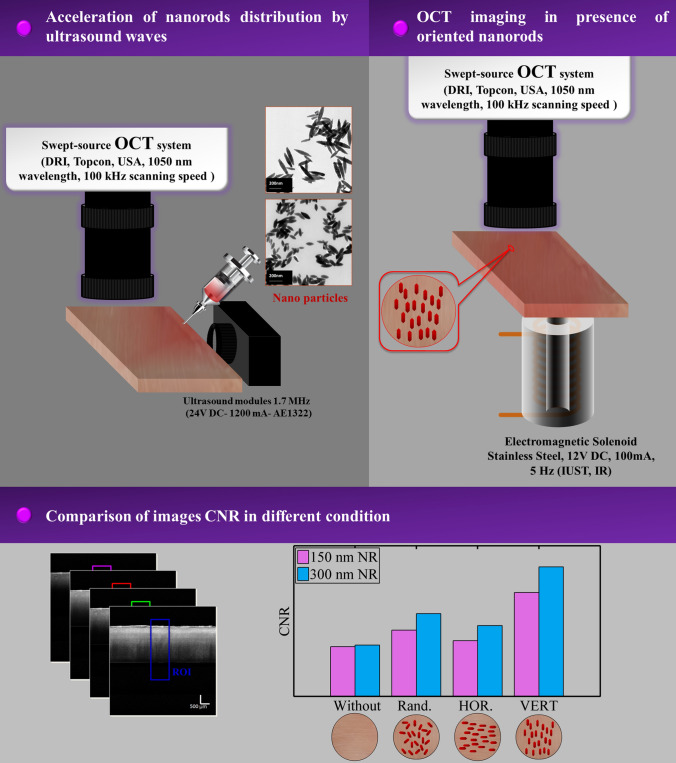

## Introduction

Optical coherence tomography (OCT) is an expanding imaging modality that has been extensively studied in recent years to overcome its inherent challenges, such as limited imaging depth and low contrast [1]. The application of nanoparticles in OCT has emerged as a prevalent strategy to enhance image contrast [2, 3]. Advances in synthetic chemistry have facilitated the production of nanoparticles with diverse morphologies and compositions, enabling their utilization across various medical imaging applications [4, 5]. Although all nanoparticle types have contributed to improvements in imaging quality, understanding how their distinct physicochemical characteristics influence image fidelity remains a critical area of research. For instance, in Ref. [2], spectral-domain OCT (SD-OCT) was combined with gold nanorods (GNRs) to achieve two-dimensional and three-dimensional imaging of live mouse embryos. Inductively coupled plasma mass spectrometry (ICP-MS) confirmed that GNRs could be effectively delivered to the embryos during ex vivo culture. The presence of GNRs resulted in enhancements in OCT signal intensity, image contrast, and penetration depth within the embryos. These findings demonstrate that GNR treatment enables more precise spatial localization and improved differentiation of organ boundaries in mouse embryos. Reference [6] provides a comprehensive review of recent advancements in optical biosensing and bioimaging techniques, focusing on three principal optical properties of gold (Au) nanoparticles: surface plasmon resonance (SPR), surface-enhanced Raman scattering (SERS), and luminescence. Additionally, it summarizes the various fabrication methods, and the optical characteristics associated with different types of Au nanoparticles. Reference [7] investigated the influence of nanorod dimensions on optical coherence tomography (OCT) signals. The findings demonstrated that larger nanorods (NRs), measuring approximately 30 × 100 nm, generate signals that are approximately 110 times more intense than those produced by conventional nanoparticles, which measure around 15 × 50 nm.

Despite these advancements, two primary challenges hinder the widespread integration of nanoparticles in OCT: the first pertains to the relatively slow diffusion and tissue penetration of nanoparticles, which limits their effective distribution within biological matrices. Typically, achieving a uniform distribution of nanoparticles within tissues post-injection requires several tens of minutes, depending on the tissue type. As reported in Ref. [8], the distribution of silver nanoparticles of various sizes within rat tissues ranged from several hours to several days. To effectively utilize nanoparticles in OCT, this time interval must be substantially reduced.

In recent years, various reports have documented the utilization of magnetic nanoparticles in OCT, referred to as magneto motive optical coherence tomography (MM-OCT). Huang et al. reported a novel magnetic Fe_3_O_4_ nanocluster that exhibits both light-scattering properties at 860 nm, suitable for OCT imaging, and absorbing capabilities at 1064 nm, which can be used for photo thermal therapy [9]. In Ref. [10], super paramagnetic iron oxide nanoparticles (SPIONs) demonstrating activity in the near-infrared (NIR) spectrum were synthesized. Owing to their exceptional optical and magnetic properties, the potential application of these environmentally friendly, green-synthesized SPIONs as exogenous contrast agents for deep-tissue MM-OCT imaging systematically evaluated ex vivo using chicken breast tissue. The second challenge pertains to the lack of control over the orientation of nanoparticles within tissues. Non-spherical nanoparticles, such as NRs, tend to adopt random orientations after injection. Since the backscattering efficiency, an essential factor in OCT, is dependent on nanoparticle orientation, this inherent randomness can hinder imaging quality.

The utilization of ultrasound waves to facilitate the distribution of optical clearing agents (OCAs) has been previously reported in Ref. [11]. The results indicated that ultrasound exposure can enhance the dispersion of oleic acid, serving as an OCA, by a factor of up to ten [11].

The application of magnetic fields and magnetic nanoparticles for targeted delivery in hyperthermia and drug delivery is an area of ongoing research. For instance, in Ref. [12], composite multilayer magnetic capsules were investigated as targeted delivery systems through both in vitro and in vivo studies under physiologic conditions. The study demonstrated the magnetic responsiveness of fluorescent composite capsules embedded with magnetite nanoparticles within the bloodstream.

This study focuses on optimizing NRs integration in biological tissues for enhanced OCT imaging. A key aspect is controlling the orientation of magnetic iron oxide NRs using a magnetic field generated by a solenoid. This alignment is crucial because the directional arrangement of NRs directly influences OCT image contrast and interpretability. By precisely orienting the NRs, we can significantly improve the clarity and diagnostic value of the OCT scans. Following this, the study also explores the use of ultrasound waves to accelerate nanoparticle distribution within tissues. The mechanical effects of ultrasound waves boost nanoparticle mobility, leading to faster and more uniform dispersion, which indirectly supports the primary goal of achieving optimal nanoparticle placement for superior OCT imaging. Subsequently, the methodology for nanoparticle preparation, experimental procedures, and the results evaluating the effects of magnetic fields and ultrasound waves on the quality of OCT images are presented.

## Materials and methods

### Sample preparation

**Fig. 1:**
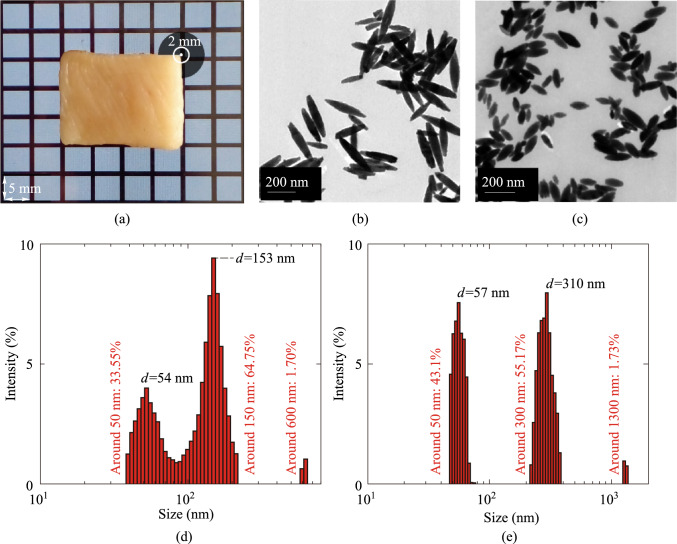
**a** Chicken breast tissue sample. **b** TEM of 300 nm NRs. **c** TEM of 150 nm NRs. **d** DLS results of 150 nm NRs. **e** DLS results of 300 nm NRs

Fresh chicken breasts, aged 45 days, were procured from a specialized poultry sales center in Tehran, Iran. Several uniform samples of the chicken breast tissue were prepared, standardizing size, shape, and weight, with dimensions of 2 cm × 1.5 cm × 0.2 cm and an average mass of 1.15 ± 0.05 g. The dimensions of the samples were determined using a caliper with an accuracy of 0.02 mm. To ensure comparability across tissue samples, all chicken breast specimens were sourced from the same supplier at the same age and weight range. Samples were consistently harvested from the Specify region of each breast. Throughout the preparation and experimental procedures, samples were maintained at 4 °C to preserve tissue integrity and minimize moisture loss. Also, preliminary OCT imaging was performed on each sample to verify tissue homogeneity and absence of gross abnormalities. Uniform orientation of muscle fibers was ensured by cutting all samples in the same direction. Moisture content was kept via storing samples in sealed laboratory containers. This rigorous standardization of sample acquisition and preparation was crucial for ensuring that observed variations in OCT results could be attributed to experimental conditions rather than inherent differences in the tissue samples. The image of one of the samples is illustrated in Fig. 1a.

### NRs preparation

Iron oxide NRs were obtained from XCHIM Company, Tehran, Iran. The choice of Fe_3_O_4_ nanoparticles over noble metal alternatives stems from their inherent magnetic characteristics, offering distinct benefits for OCT applications. The ability to magnetically orient Fe_3_O_4_ provides dynamic controllability over contrast enhancement, facilitating repeatable imaging sessions and directional amplification of scattering signals. This level of control and specificity is crucial for advancing OCT’s capabilities in tissue imaging. The synthesis of these NRs was conducted following the methodologies outlined in Refs. [13, 14]. Synthesis of magnetite (Fe_3_O_4_) NRs via a One-Step Hydrothermal and Reduction Process are described in 7 steps (a − g) as follows:

- Preparation of Precursor Solution: A homogeneous solution was prepared by dissolving 6.0 mmol/L of Ferric chloride hexahydrate (FeCl_3_·6H_2_O) with 99% purity in 60 mL of deionized water (DI water). The solution was subjected to continuous stirring (500 r/min) for 30 min to ensure complete dissolution.
- Hydrothermal Reaction: The prepared FeCl_3_·6H_2_O solution was transferred into a Teflonlined stainless-steel autoclave. The reaction was carried out under hydrothermal conditions at a temperature of 100 °C for either 7 h or 12 h. These varying hydrothermal durations were employed to yield precursors resulting in final Fe_3_O_4_ NRs of approximately 300 nm (aspect ratio ~ 6) and 150 nm (aspect ratio ~ 3), respectively.
- Precursor Collection and Purification: Following the hydrothermal treatment, the resulting β-FeOOH precursors were collected via centrifugation at 7000 r/min for 30 min. The collected precursors were subsequently purified through washing with ethanol and DI water, followed by drying at 60 °C.
- Preparation for Reduction Reaction: In the subsequent step, 20 mg of the size-fractionated β-FeOOH NRs were uniformly dispersed in 6 mL of trioctylamine (TOA, Hosea Chem, China). To this mixture, 200 μL of oleic acid (OA) were added, acting as a capping agent. The resulting solution was stirred (at 500 r/min) for 2 h to achieve a homogeneous dispersion and ensure adequate surface functionalization of the precursors. The mixture appeared as a yellow gelatinous substance.
- Collection of Functionalized Precursors: The yellow gelatinous mixture was collected by centrifugation at 7500 r/min for 15 min.
- Reduction Reaction: The prepared, functionalized precursors were then subjected to a reduction reaction. This was conducted within a tube furnace under a controlled atmosphere, specifically a flow of mixed gas consisting of 90% Ar and 10% H_2_. The reduction temperature was maintained at 300 °C for a duration of 1.5 h. Following the reaction, the furnace was allowed to cool naturally to room temperature.
- Final Product Purification and Drying: The synthesized Fe_3_O_4_ NRs were further purified through size fractionation using centrifugation. The collected NRs were thoroughly washed with hexane to remove any residual impurities or unreacted capping agents. Finally, the Fe_3_O_4_ NRs were dried at 60 °C to ensure complete evaporation of the washing solvent.

The transmission electron microscopy (TEM) image of the synthesized NRs is presented in Fig. 1b and c. The results of the Dynamic Light Scattering (DLS) test, which was performed using a Chinese DLS system (Model: LS-N009, Test Range: 1–1000 nm, Main Laser: 532 nm semiconductor laser, scattering angle: 90°, Manufacturer: Changsha Langshuo Company, China), are presented in Fig. 1d and e. According to the results of the DLS analysis, the hydrodynamic sizes of Fe_3_O_4_ NRs were 54 nm and 153 nm for short NRs, and 57 nm and 310 nm for long NRs. These peaks not only indicate the length and width of the NRs but also represent their rotational diffusion. In conclusion, a suspension of Fe_3_O_4_ NRs in water, with a concentration of 5 mg/mL for both sizes of the NRs, was prepared and ready for injection into the tissue. Although different studies have investigated the toxicity of iron oxide nanoparticles, their safe application at limited doses has led to their widespread use in various medical applications [9, 10].

### Solenoid and ultrasound modules

A solenoid was employed to orient the NRs within the tissue, which was designed and fabricated by the Science and Industry Workshop Center, Tehran, Iran (12 V DC, 100 mA, 18 mT, length: 3 cm, diameter: 1.5 cm). Prior to each OCT imaging session, a magnetic field must be applied to the tissue to ensure that the NRs are aligned in the desired orientation.

**Fig. 2:**
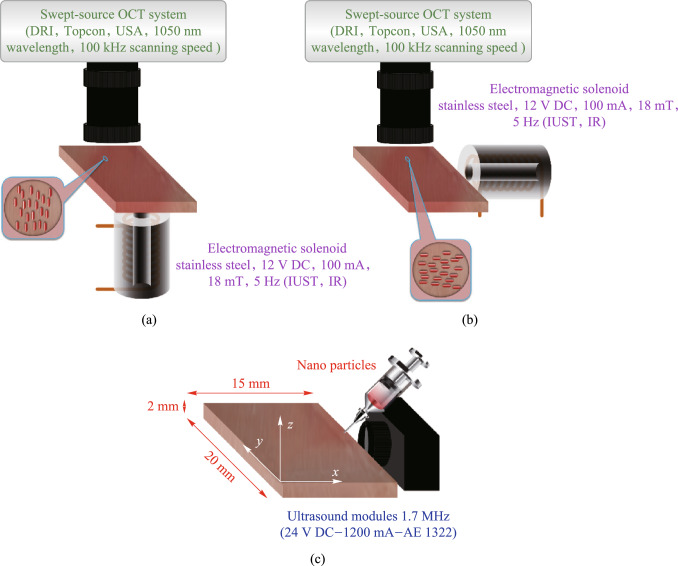
Schematic of the experimental test: OCT in the presence of **a** vertical and **b** horizontal NRs. **c** Schematic of NRs injection and application of ultrasound waves

The natural self-distribution of nanoparticles after tissue injection can be time-consuming. To overcome this, our study utilizes a 1.7 MHz continuous ultrasound module (24 V DC, 1200 mA, AE1322) to accelerate NR distribution within the tissue. After the injection of the NRs, we first expedite the distribution process for 5 min using ultrasound radiation, and subsequently, we orient the NRs in the desired direction by applying a magnetic field. Figure 2 provides a schematic diagram of the experimental test described. OCT imaging in this study was performed using Swept-Source OCT system (DRI, Topcon, USA, 1050 nm wavelength). The effect of several factors on OCT images has been investigated. The first factor is the effect of the same orientation of the NRs in the OCT images. Consequently, horizontal and vertical polarizations were applied to the nanoparticles, as schematically illustrated in Fig. 2a and b.

As depicted in Fig. 2c, injection of Fe_3_O_4_ suspension in water with a concentration of 5 mg/mL is performed from the top-surface of the tissue at coordinates (*x* = 15 mm, *y* = 10 mm, *z* = 2 mm) and at an angle of 45º such that the nanoparticles are given an initial velocity toward the bottom-left. The distance from the injection site to the imaging region is approximately 8 mm, and the imaging region is a square of 6 mm × 6 mm. The ultrasound module irradiates ultrasound waves from the right lateral surface toward the left. This arrangement facilitates rapid distribution of nanoparticles toward the imaging area immediately after injection.

It is worth noting that the ultrasound module can be employed for ex vivo imaging applications in any direction desired for nanoparticle distribution. However, for in vivo imaging, nanoparticle injection is only feasible through the top surface of the tissue; consequently, the ultrasound module must also irradiate the top surface. Published reports have demonstrated that injecting nanoparticles from the top surface and subsequently applying ultrasound waves from the same surface can significantly enhance the rate of nanoparticle distribution [11].

### CNR and SNR calculation

The parameter of contrast-to-noise ratio (CNR) in OCT images is defined as follows [15]:

1 $${\text{CNR}} = \frac{1}{M}\mathop \sum \limits_{m = 1}^{M} \frac{{\left( {\mu_{m} - \mu_{b} } \right)}}{{\sigma_{m}^{2} + \sigma_{b}^{2} }},$$

where *μ*_***m***_ and *σ*_***m***_ in CNR represent the mean and standard deviation of the *m*th region of interest in OCT signal, respectively, and *μ*_***b***_ and *σ*_***b***_ are the mean and standard deviation of the background noise region in OCT signal, respectively.

Moreover, SNR quantifies the strength of the backscattered signal relative to the background noise. The SNR in logarithmic decibels scale is calculated as follows [1]:

2 $${\text{SNR}}_{{{\text{dB}}}} = 10\log_{10} \frac{{I_{{{\text{signal}}}} }}{{I_{{{\text{noise}}}} }},$$

where *I*_signal_ represents the mean signal intensity and *I*_noise_ denotes the mean noise intensity. *I*_signal_ is estimated from the mean intensity within a distinct anatomical or pathological parts of ROI, while *I*_noise_ is often derived from the variance of intensity in a background parts of ROI [1].

## Results and discussion

**Fig. 3:**
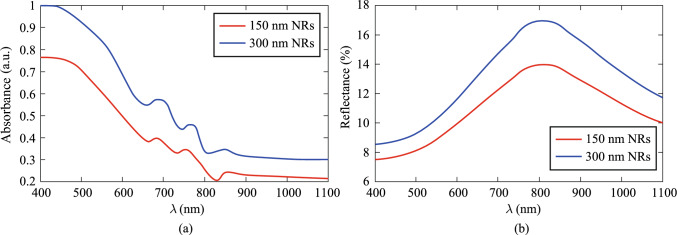
Nanorods optical properties: **a** absorbance and **b** reflectance of NRs

As an initial step, the absorption and reflectance properties of the NRs were examined to assess their efficacy in OCT. These parameters were determined by a custom modified Double Beam UV–Vis Spectrophotometer (UV-2100, BRAIC, China). Tungsten source is used in this system, and its working wavelength range is from 400 nm to about 1100 nm. Figure 3a and b illustrate the absorbance and reflectance spectra of the synthesized NRs, respectively. The observed low absorption coupled with relatively high reflectance near 1050 nm, the operational wavelength of the OCT system, suggests these nanoparticles effectively enhance backscatter signals, thereby potentially improving OCT image quality. It is important to highlight that the aspect ratio of the NRs significantly influences plasmon resonance (PR) behavior. As reported in Ref. [16], an increase in the aspect ratio shifts the plasmon resonance wavelength toward longer values. The enhanced absorption and reflectance observed in the 300 nm NRs relative to the 150 nm NRs can be attributed to size-dependent plasmonic effects governing both absorption and scattering. As documented in multiple studies, including Ref. [6], which have examined the absorbance (*A*), reflectance (*R*), and transmittance (*T*) of nanoparticle-containing materials, larger nanoparticles typically exhibit increased absorption and reflectance. Consequently, in accordance with the fundamental relationship *A* + *R* + *T* = 1, an augmentation in both absorbance and reflectance leads to a concomitant reduction in transmittance.

The objective of this study is to examine the influence of NRs orientation on OCT imaging. To achieve this, prepared NRs were injected into sliced chicken breast tissue. In the initial phase of the experiments, the ultrasound module was not employed to facilitate NRs distribution. Instead, a 45-min period was allotted for the NRs to attain a uniform dispersion within the tissue. Subsequently, magnetic fields were applied to align the NRs, and OCT imaging was performed. It is crucial to note that the alignment process of the NRs under a magnetic field is rapid, taking less than one minute. This phenomenon has been investigated in previous studies (for example, Ref. [17]), which experimentally measured the duration required for NR orientation under magnetic influence. The findings indicate that NRs rapidly align with the applied magnetic field. This consideration is significant, as prolonged exposure to the magnetic field can cause the NRs to migrate toward the solenoid’s attractor, disrupting the uniform distribution of nanoparticles within the tissue. Such migration results in accumulation near the solenoid are used for drug delivery and hyperthermia treatment applications [18].

**Fig. 4:**
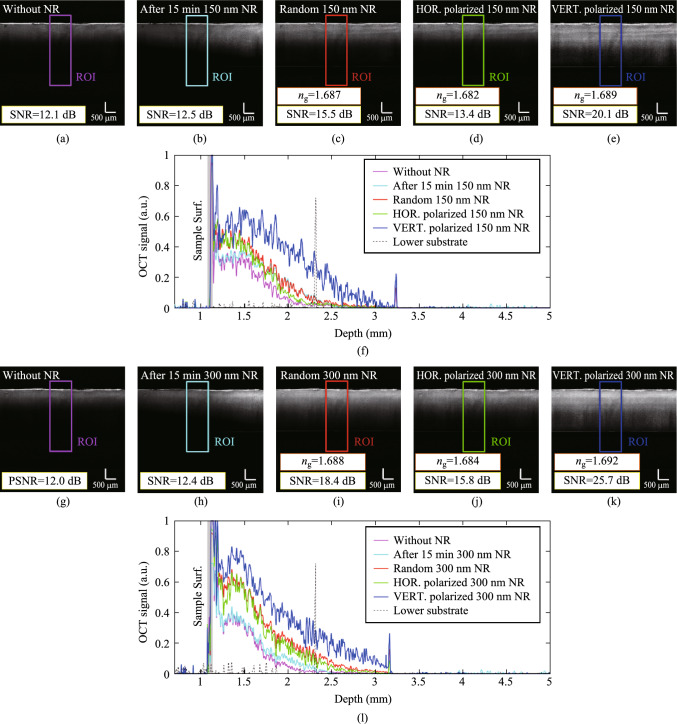
OCT images of chicken breast tissue **a**, **g** without NRs, and 15 min post-injection of **b** 150 nm NRs and **h** 300 nm NRs, and random orientation of **c** 150 nm and **i** 300 nm NRs, and for horizontally orientation of **d** 150 nm and **j** 300 nm NRs, and for vertically orientation of **e** 150 nm and **k** 300 nm NRs. Also, **f** and **l** are ROIs OCT signal for 150 nm NRs and 300 nm NRs, respectively

Figure 4 presents OCT images obtained under various conditions. Figure 4a and g depict chicken breast tissue without NRs. Figure 4b and h illustrate the random distribution of NRs 15 min post-injection. The NRs enter the Region of Interest (ROI) approximately 15 min post-injection, subsequently influencing the CNR and SNR parameters. Figures 4c (random state of NRs), 4d (horizontal state of NRs) and 4e (vertical state of NRs) were captured in the presence of 150 nm NRs, while Fig. 4i (random state of NRs), 4j (horizontal state of NRs) and 4k (vertical state of NRs) involved 300 nm NRs. In all cases involving NRs, a 45-min period was allotted for their distribution within the tissue. Previous studies have reported the arrival time of uniformly distributed nanoparticles ranging from a few minutes to several hours [8–10]. However, based on testing multiple samples, we have determined that 45 min is the optimal duration for the effective distribution of iron oxide NRs within tissue. Figure 4f and l correspond to signals from designated ROI.

Figure 4 reveals several noteworthy findings. First, under similar conditions, 300 nm NRs enhanced SNR by at least 18% more than 150 nm NRs, consistent with prior research indicating larger NRs provide superior OCT enhancement [7]. Second, the highest contrast was observed in Fig. 4d and i, where NRs were vertically aligned. In contrast, random and horizontally aligned NRs demonstrated lesser effects. This aligns with literature indicating that the optical scattering and absorption properties of NRs are orientation dependent [18]. Ultimately, the greatest enhancement in SNR, up to a twofold increase relative to nanoparticle-free tissue—was achieved with vertically oriented 300 nm NRs. An additional valuable parameter that can be derived from OCT images is the tissue’s group refractive index (*n*_g_). To measure *n*_g_, initially, OCT images are acquired of the lower substrate (glass surface) in the absence of tissue. The resulting OCT image reveals a peak corresponding to the glass surface, which shifts depending on whether the tissue sample is present or absent. Key measurements for determining the refractive index include the optical shift (*OS*), which denotes the displacement of the peak, and the optical thickness (*OT*), representing the distance between the surface and bottom peaks. Utilizing the method introduced in Ref. [1], *n*_g_ is obtained from *n*_g_ = *OT*/(*OT − OS*). The *n*_g_ for the samples in Fig. 4 was determined using the same method. However, in Fig. 4a and f, calculation was not possible due to unclear lower substrate peaks. Due to subtle differences in the final peak depth of the different states in Fig. 4f and l, the *OS* and *OT* intervals will differ, consequently leading to a difference in their group refractive indices. Utilizing the principles of error propagation and considering the relative errors associated with the *OT* and *OS* parameters, the calculated group refractive index exhibits an error of ± 0.002. The highest group refractive index was observed in the vertical NRs, with refractive indices of *n*_g_ = 1.689 for 150 nm NRs and *n*_g_ = 1.692 for 300 nm NRs. This was followed by the NRs in a random orientation, with *n*_g_ = 1.687 for 150 nm and *n*_g_ = 1.688 for 300 nm NRs. The lowest values were recorded for the horizontal NRs, with *n*_g_ = 1.682 for 150 nm and *n*_g_ = 1.684 for 300 nm NRs. Analyzing changes in refractive index may significantly improve tissue characterization and tumor detection. By measuring these indicators, OCT makes it possible to distinguish between healthy and diseased tissues. Determining the group refractive index is therefore a crucial and very advantageous task in medical applications. For instance, using the same methods described in Ref. [1], the group refractive index of chicken breast tissue was found to be 1.410. The influence of nanorod presence on tissue refractive index is demonstrated by the roughly 0.2 difference in the group refractive index of chicken breast tissue in the presence and absence of Fe_3_O_4_ NRs. This technique can also be used to find other tissue heterogeneities.

Additionally, the variation in the group refractive index across different NRs polarizations may be attributed to the fact that, during propagation through the tissue, the light interacts partially with the host tissue and partially with the NRs. Changes in the NRs’ polarization alter the proportion of light interacting with the NRs. For instance, when the NRs are oriented vertically, the light interacts more extensively with the NRs along the propagation path, leading to an increase in the group refractive index.

NRs, anisotropic nanostructures with distinct longitudinal and transverse dimensions, exhibit unique optical properties primarily governed by surface plasmon resonance (SPR), which arises from the collective oscillation of free electrons in metallic nanoparticles. Their elongated geometry leads to anisotropic scattering, where scattering intensity and directionality are highly dependent on the nanorod’s orientation relative to the incident light’s electric field; vertical alignment strongly excites longitudinal plasmons, significantly enhancing scattering and absorption at specific resonance wavelengths, while horizontal alignment primarily excites lower-energy transverse plasmons, resulting in diminished optical effects. This SPR phenomenon, particularly the backscattering enhancement observed in vertically aligned nanorods, is crucial for improving SNR and CNR in OCT. SPR properties’ dependence on nanorod orientation has been investigated in several papers. For example, Ref. [19] demonstrates a polarization-sensitive photothermal imaging technique to determine the orientation of single gold nanorods by exploiting the polarization dependence of their transverse and longitudinal surface plasmon resonances. The method offers high accuracy, verified by SEM, and uniquely allows orientation determination from the transverse mode, which is insensitive to aspect ratio and medium refractive index. Furthermore, the aspect ratio of NRs critically influences their plasmon resonance wavelength and scattering intensity, with longer nanorods typically supporting longitudinal plasmons at longer wavelengths and displaying stronger scattering, thus enabling optical tunability for specialized applications.

**Fig. 5:**
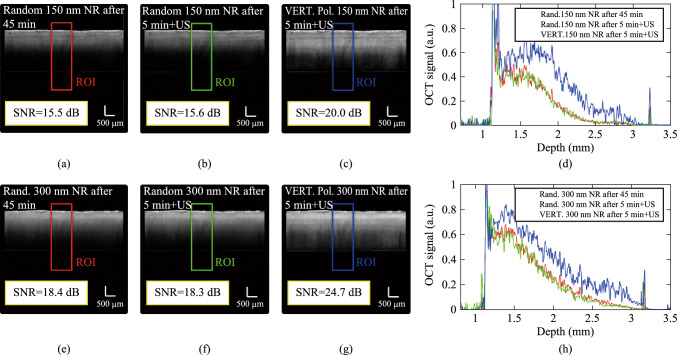
Reduced NRs distribution time. OCT images after 45 min spontaneous distribution of **a** 150 nm and **e** 300 nm NRs and after 5 min applying ultrasound waves **b** 150 nm and **f** 300 nm NRs and after 5 min applying ultrasound waves with vertical magnetic field for **c** 150 nm and **g** 300 nm NRs. Also, **d** and **h** are ROIs OCT signals for 150 nm and 300 nm NRs, respectively

Figure 5 displays OCT images used to assess the effect of ultrasound waves on the dispersion of NRs within tissue samples. In this experiment, two chicken breast tissue samples were injected with 150 nm and 300 nm NRs. Following injection, the samples were allowed for 45-min period to facilitate uniform distribution of the NRs. OCT images captured under these conditions are presented as Fig. 5a and e. The experiment was then repeated using identical NR injections in two additional tissue samples; however, in this instance, ultrasound irradiation was applied for 5 min during the process, as illustrated in Fig. 2c, to enhance NR mobility within the tissue OCT images corresponding to these conditions are shown as Fig. 5b and f. In these cases, the distribution of NRs in the tissue is random. Finally, we conducted repeat experiments on two additional samples, incorporating both ultrasound waves and the application of a magnetic field to induce vertical alignment of the NRs. The results can be seen in Fig. 5c and g. Figure 5d and h illustrate the OCT signals within the ROIs.

The acoustic radiation force exerted by ultrasound waves can actively propel nanorods, facilitating their distribution within tissues and significantly decreasing the time required for this process. Recent scholarly articles have introduced the application of ultrasound waves for the precise guidance and distribution of nanoparticles. Reference [20] introduces ultrasound-activated micro-robots, composed of lipid-shelled microbubbles, capable of autonomous aggregation and propulsion, highlighting the critical role of ultrasound in their guided navigation. These micro-robots demonstrate in vivo navigation within cerebral vasculature, successfully achieving upstream motion against a blood flow of approximately 10 mm/s, marking a significant advancement for targeted therapeutic delivery in the brain. Beyond nanoparticles, ultrasound waves accelerate the distribution of any dispersible material. As documented in Ref. [11], optical clearing agents were distributed 10 times faster in the human skin by continuous sonication (1 MHz, 2 W/cm^2^, 1–5 min), a result comparable to our findings. Also, oleic acid and ultrasound allowed increasing OCT signal amplitude up to 3.3-fold with more than twice improved depth penetration during 30 min in the human skin. The efficacy of the ultrasound module in regulating the spatial distribution of foreign material (clearing agent or nanoparticles) within the tissue is critically determined by the applied ultrasonic wave intensity. Specifically, enhanced intensity facilitates a demonstrably greater impact.

The expedited nanoparticle distribution observed, reducing the time from 45 to 5 min, is attributed to the multifaceted effects of ultrasound on tissue. Specifically, acoustic streaming, a phenomenon involving fluid movement induced by sound waves, plays a significant role in enhancing the convective transport of nanoparticles within the tissue matrix. Furthermore, the mechanical agitation generated by ultrasound contributes to improved penetration by disrupting local barriers and facilitating the movement of nanoparticles through interstitial spaces. These mechanisms collectively enhance the diffusivity and penetration depth of the nanoparticles. The intensity of ultrasound produced by the ultrasound module is approximately 4 W/cm^2^. Given that this intensity is in the standard range for commercial diagnostic systems (1–10 W/cm^2^) [11], such as sonography systems, there is no concern regarding tissue ablation. Furthermore, OCT images did not indicate any damage or change in tissue state following the application of the ultrasound waves. Also, prior to in situ application, the Fe_3_O_4_ NRs were subjected to the specified ultrasound parameters using the identical module. Subsequent morphological characterization via TEM confirmed the structural integrity of the NRs, showing no evidence of deformation or fracture after this initial sonication process.

Two notable findings emerge from Fig. 5. First, the application of ultrasound waves during a 5-min irradiation period resulted in OCT contrast levels comparable to those observed after a 45-min period without ultrasound. This indicates that ultrasound waves significantly expedite nanoparticle distribution within tissue, approximately ninefold faster, thus markedly reducing the time required for effective nanoparticle dispersion in optical imaging procedures. Secondly, the SNR obtained from 150 nm NRs following ultrasound application exceeded that achieved after 45 min without ultrasound, highlighting improved nanoparticle distribution speed for the smaller NRs. Conversely, this enhancement was not observed for 300 nm NRs, suggesting that the distribution rate of larger NRs remains comparatively slower, consistent with findings reported in Refs. [21, 22].

**Fig. 6:**
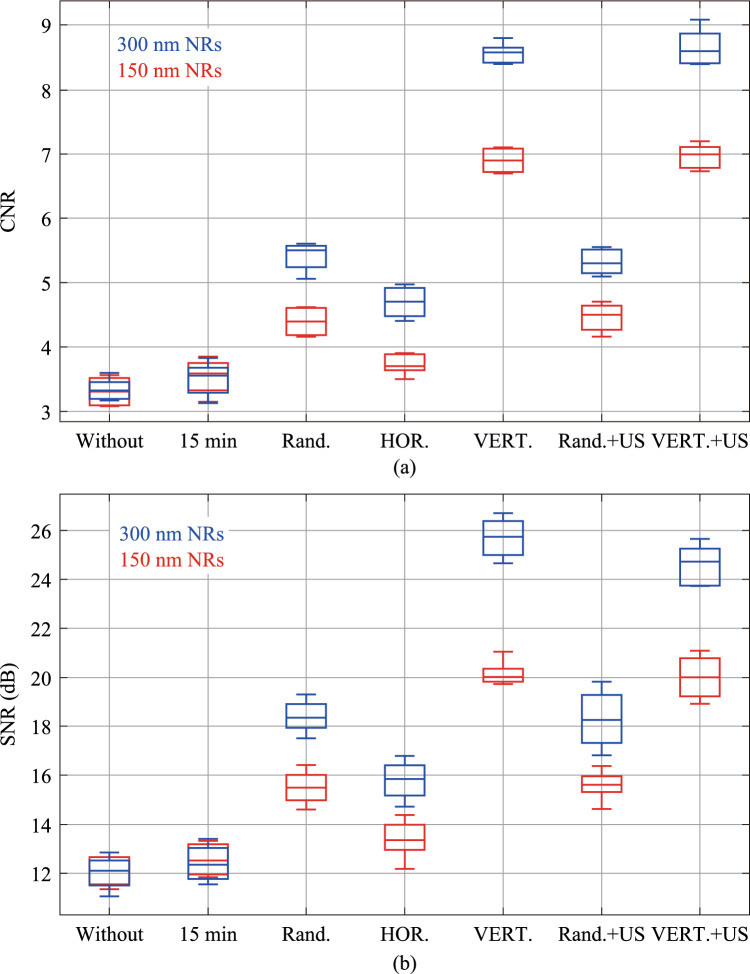
**a** CNR of OCT images, for each CNR, five OCT images were used and a one-way ANOVA with Bonferroni post-hoc test was performed (*p* < 0.03) which shows that different groups of results are significantly different from each other. **b** SNR of OCT images, for each SNR, five OCT images were used and a one-way ANOVA with Bonferroni post-hoc test was performed (*p* < 0.05). Red line: 150 nm NRs and blue line: 300 nm; Without: without the use of NRs; 15 min: 15 min post-injection of NRs; Rand: random state of NRs after 45 min; HOR: horizontal orientation of NRs; VERT: vertical orientation of NRs; US: using ultrasound waves for 5 min

The CNR of the OCT images were evaluated under varying conditions (Fig. 6a). The most significant finding revealed that applying a vertical magnetic field resulted in more than a twofold increase in image contrast compared to the tissue without nanoparticles. Figure 6b also presents the SNR results of OCT images under various conditions. Furthermore, an ANOVA with Bonferroni post-hoc analysis was performed on the results, the details of which are provided in the figure caption.

As depicted in Fig. 6a, the CNR demonstrated an approximate 160% improvement, ranging from its lowest value in nanoparticle-free tissue (approximately 3.3) to its peak value observed with vertically polarized 300 nm nanorod (approximately 8.6). Furthermore, vertically polarized nanorods generated approximately 60% better contrast compared to randomly oriented NRs. Collectively, these findings underscore the efficacy of the proposed method in enhancing the CNR of OCT images. Also, quantitative analysis of the SNR in Fig. 6b, reveals the following results: The lowest SNR is observed in the absence of nanorods. Specifically, the SNR of OCT images of tissue without any contrast agent is approximately 12 dB. Upon injection and distribution of nanorods within the tissue to achieve uniform distribution, the SNR increases. For instance, 15 min post-injection of 150 nm and 300 nm nanoparticles, the SNR shows an increase of 3% and 4%, respectively. Following uniform distribution, if the nanorods are oriented horizontally using an external magnetic field, the SNRs increase by 10% and 31%, respectively. If the nanorods exhibit random orientation, an increase of 28% and 53% is observed. Finally, when the nanorods are vertically aligned, the SNR experiences a substantial increase of 66% and 114%, respectively. The amount of SNR increase achieved in the vertical 300 nm NRs configuration is comparable to the SNR increase reported for OCT images in Ref. [1] for the case of 30 min post-injection of glycerol as a clearing agent to chicken breast tissue. The comprehensive results pertaining to the mean CNR and mean SNR of OCT images are systematically compiled in Table 1. A notable observation in the table is that the vertical polarization state of the nanorods exhibits a higher SNR compared to the combined state of vertical polarization and ultrasound waves. This phenomenon may be related to the disorientation of the vertically oriented NRs induced by the ultrasound waves.

**Table 1:** CNR and SNR of chicken breast OCT images in different conditions

	CNR		SNR	
	150 nm	300 nm	150 nm	300 nm
Without NR	3.31	3.34	12.12	11.89
15 min post-NR injection	3.54	3.49	12.54	12.41
Random NR	4.40	5.40	15.51	18.43
Horizontal NR	3.73	4.70	13.38	15.83
Vertical NR	6.90	8.56	20.14	25.69
Random NR + Ultrasound	4.45	5.38	15.59	18.28
Vertical NR + Ultrasound	6.96	8.66	19.98	24.65

## Conclusion

This study examined the effects of NR orientation within tissue on OCT imaging by integrating a magnetic field into the imaging setup and employing iron oxide NRs. Three orientation modes, random, vertical, and horizontal, were examined. The results indicate that, in the most optimal scenario, the CNR and SNR of images acquired with vertically oriented NRs exhibited more than a twofold increase compared to those obtained without NRs. Additionally, the use of ultrasound waves reduced nanoparticle distribution time within tissue by up to nine times. While the ultrasound module offers considerable flexibility for in vitro applications, allowing for the application of acoustic waves along a desired vector to induce the controlled distribution of NRs within the tissue sample, its utility in in vivo settings is subject to a significant directional constraint. Specifically, in vivo applications are often limited to transdermal delivery, restricting the ultrasound waves to a superior (top-down) trajectory relative to the targeted tissue. This inherent limitation dictates and restricts the available methodologies for effectively accelerating or spatially guiding the distribution of NRs deep within the biological environment. Additionally, the group refractive index of the tissue in various polarization states of the NRs was quantified through OCT imaging. The findings indicated that the group refractive index attained its maximum value in the vertical polarization state of the NRs. It is noteworthy that, in general, the group refractive index of biological tissues tends to exceed their corresponding phase refractive index [23]. The findings presented herein establish a significant methodological advance for enhancing the sensitivity and speed of OCT-based imaging. Crucially, the combination of magnetic manipulation and ultrasound-assisted delivery validates this system’s strong potential for in vivo deep-tissue OCT imaging, magnetic targeting applications, and real-time functional imaging. A short outlook on clinical or translational feasibility suggests that our demonstrated safety profile (ultrasound intensity below ablation thresholds) and improved contrast metrics provide a solid foundation for transitioning these integrated OCT methodologies from the experimental setting toward standard clinical practice. Therefore, the findings and achievements of this paper promise significant advancements across dermatological diagnostics, dental imaging for periodontal assessment, neuroimaging applications in cognitive function analysis, vascular evaluation, and in vitro tissue heterogeneity mapping. This synergistic enhancement of OCT quality directly translates to improved diagnostic accuracy and deeper structural insight in these diverse biomedical fields.

## Data Availability

The data that support the findings of this study are available from the corresponding author, upon reasonable request.
